# Removal of Transmissible Spongiform Encephalopathy Prion from Large Volumes of Cell Culture Media Supplemented with Fetal Bovine Serum by Using Hollow Fiber Anion-Exchange Membrane Chromatography

**DOI:** 10.1371/journal.pone.0122300

**Published:** 2015-04-13

**Authors:** Ming Li Chou, Andy Bailey, Tiffany Avory, Junji Tanimoto, Thierry Burnouf

**Affiliations:** 1 Graduate Institute of Medical Science, College of Medicine, Taipei Medical University, Taipei, Taiwan; 2 ViruSure, Tech Gate Science and Technology Park, Donau City Strasse 1, A-1220, Vienna, Austria; 3 Asahi Kasei Medical Co, Ltd., Tokyo, Japan; 4 Graduate Institute of Biomedical Materials and Tissue Engineering, College of Oral Medicine, Taipei Medical University, Taipei, Taiwan; Nagasaki University Graduate School of Biomedical Sciences, JAPAN

## Abstract

Cases of variant Creutzfeldt-Jakob disease in people who had consumed contaminated meat products from cattle with bovine spongiform encephalopathy emphasize the need for measures aimed at preventing the transmission of the pathogenic prion protein (PrP^Sc^) from materials derived from cattle. Highly stringent scrutiny is required for fetal bovine serum (FBS), a growth-medium supplement used in the production of parenteral vaccines and therapeutic recombinant proteins and in the ex vivo expansion of stem cells for transplantation. One such approach is the implementation of manufacturing steps dedicated to removing PrP^Sc^ from materials containing FBS. We evaluated the use of the QyuSpeed D (QSD) adsorbent hollow-fiber anion-exchange chromatographic column (Asahi Kasei Medical, Tokyo, Japan) for the removal of PrP^Sc^ from cell culture media supplemented with FBS. We first established that QSD filtration had no adverse effect on the chemical composition of various types of culture media supplemented with 10% FBS or the growth and viability characteristics of human embryonic kidney (HEK293) cells, baby hamster kidney (BHK-21) cells, African green monkey kidney (Vero) cells, and Chinese hamster ovary (CHO-k1) cells propagated in the various culture-medium filtrates. We used a 0.6-mL QSD column for removing PrP^Sc^ from up to 1000 mL of Dulbecco’s modified Eagle’s medium containing 10% FBS previously spiked with the 263K strain of hamster-adapted scrapie. The Western blot analysis, validated alongside an infectivity assay, revealed that the level of PrP^Sc^ in the initial 200mL flow-through was reduced by 2.5 to > 3 log_10_, compared with that of the starting material. These results indicate that QSD filtration removes PrP^Sc^ from cell culture media containing 10% FBS, and demonstrate the ease with which QSD filtration can be implemented in at industrial-scale to improve the safety of vaccines, therapeutic recombinant proteins, and ex vivo expanded stem cells produced using growth media supplemented with FBS.

## Introduction

Pathogenic prions are protein particles composed of the misfolded isoform of a naturally occurring nonpathogenic cellular glycoprotein, known as the prion protein (PrP^C^). The misfolded prion protein (PrP^Sc^) causes prion disease. Prion diseases are progressive, ultimately fatal neurodegenerative disorders classified as transmissible spongiform encephalopathies (TSEs). Prion diseases affect both humans and animals. In humans, several forms of TSE have been identified, including Creutzfeldt-Jakob disease (CJD), kuru, Gerstmann-Sträussler-Scheinker syndrome, and fatal familial insomnia [1]. In animals, the most notable TSE is bovine spongiform encephalopathy (BSE).

The BSE epidemic, which occurred in the United Kingdom and later spread to Europe, was caused by the use of the carcasses of cows with BSE in commercial cattle feed. Infectivity was subsequently passed to humans who consumed meat products from cattle that had fed on the contaminated cattle feed, causing a variant of CJD (vCJD) [2,3]. Inadvertent transmission of the PrP^Sc^ to humans, through contaminated therapeutic blood products, biologicals, tissues, and surgical treatments, has also occurred [4–6], revealing that parenteral administration is an effective route for prion transmission. Similar transmissions have been observed in veterinary vaccine products using Louping-ill vaccine preparations inadvertently prepared from Scrapie infected brain preparations [4].

The risk of the transmission of prions through the use of therapeutic biologicals is therefore a major concern to regulatory authorities worldwide. Highly stringent scrutiny is required for fetal bovine serum (FBS), which is still widely used in the pharmaceutical and biotechnology industries as a supplement for cell culture media in the production of therapeutic recombinant proteins and vaccines, the expansion of stem cells for regenerative medicine, and the storage of transplant tissues [7–10]. The use of alternatives to FBS and the production of FBS from the fetuses of cows raised in geographical areas free of BSE have been proposed as means of reducing the risk of bovine PrP^Sc^ transmission [11]. The safety of materials produced using FBS can be improved by the implementation of manufacturing steps aimed at eliminating prion infectivity without diminishing product quality. This is routinely evaluated in partitioning processes for blood products using the protease resistant PrP^Sc^ protein as a marker to monitor the removal of infectious prions. All studies performed to date have demonstrated a close correlation between removal of the PrP^Sc^ marker and the removal of infectivity [12–14]. We therefore selected to monitor the partitioning of PrP^Sc^ in the investigations reported here. Because PrP^Sc^ is resistant to inactivation conducted using physicochemical methods [15], inactivating PrP^Sc^ without inadvertently altering the components of FBS that support cell growth might be difficult. Therefore, we hypothesized that removing PrP^Sc^ from FBS by using chromatography was more readily achievable because a previous study reported that subcellular fractions of scrapie-affected mouse brain demonstrated reduced scrapie activity, indicating that physically separating the PrP^Sc^ from other biological materials might be possible [16]. We employed a novel adsorbent hollow-fiber anion-exchange chromatographic column to remove PrP^Sc^ from cell culture media supplemented with FBS through the selective binding of PrP^Sc^ to the adsorbent hollow-fiber membrane [17].

## Materials and Methods

### Cell cultures tests

#### Growth media

Gibco cell culture reagents (Invitrogen, Carlsbad, CA, USA) were used exclusively in the cell culture experiments. Dulbecco’s modified Eagle’s medium (DMEM) was supplemented with 10% FBS, 0.1 mM nonessential amino acids, and 1 mM sodium pyruvate. Minimum essential medium (MEM) was supplemented with 10% FBS, 2.2 g of NaHCO_3_, and 10 mL/L of 100× solutions of L-glutamine, penicillin-streptomycin, sodium pyruvate, and nonessential amino acids. The F-12K medium was supplemented with 10% FBS.

#### QyuSpeed D chromatography of growth media containing 10% FBS

The chromatographic experiments were performed at ambient temperature (20°C ± 2°C). A QyuSpeed D (QSD) adsorbent hollow-fiber anion-exchange chromatographic column (Asahi Kasei Medical, Tokyo, Japan) was used according to the manufacturer instructions [17]. Filtration was performed using an ÄKTA Prime chromatographic system (GE Healthcare, Waukesha, WI, USA) with a P960 chromatography pump operated at a flow rate of 3.0 ± 0.3 mL/min and a maximal transmembrane pressure of 0.2 MPa. Pressure, conductivity, and absorbance at 280 nm were monitored throughout the experiment. The QSD column was equilibrated with phosphate-buffered saline (PBS) by the passage of 12 mL of PBS (pH 6.0), followed by 20 mL of PBS (pH 7.3), at 3 mL/min. One liter of growth medium supplemented with 10% FBS was passed through a 0.2 μm filter (Thermo Fisher Scientific, Waltham, MA, USA) before being passed through the QSD column. The filtrate was collected in three 300-mL fractions (FT1, FT2, and FT3). The column was washed with the equilibration buffers to remove the unbound proteins from the anion-exchange membrane until the absorbance of the filtrate returned to the baseline level. The column was eluted with 1 M NaCl-PBS buffer (pH 7.3) to remove the adsorbed proteins. Samples were taken at all stages for characterization. The FT1, FT2, and FT3 fractions were analyzed separately or as pools (FT1+FT2 or FT1+FT2+FT3 at equivalent volumes) before being used in the cell culture experiments.

#### Sodium dodecyl sulfate-polyacrylamide gel electrophoresis

The protein content of culture medium samples was analyzed using sodium dodecyl sulfate-polyacrylamide gel electrophoresis (SDS-PAGE) under nonreducing and reducing conditions, as previously described [18], by using acrylamide gradient gels, reagents, and an electrophoretic system purchased from Invitrogen. Novex Sharp prestained protein molecular-weight standards (Invitrogen) were used for assessing the molecular mass of proteins in the samples.

#### Chemical analysis

The levels of glucose, chloride, sodium, potassium, calcium, phosphate, magnesium, iron, total iron capacity, ferritin, vitamin B12, and folate in the growth medium samples were quantified using a Roche MODULE P800 Automatic Biochemical Analyzer (Roche Diagnostics, Indianapolis, IN, USA).

#### Cell culture conditions

**Fig 1:**
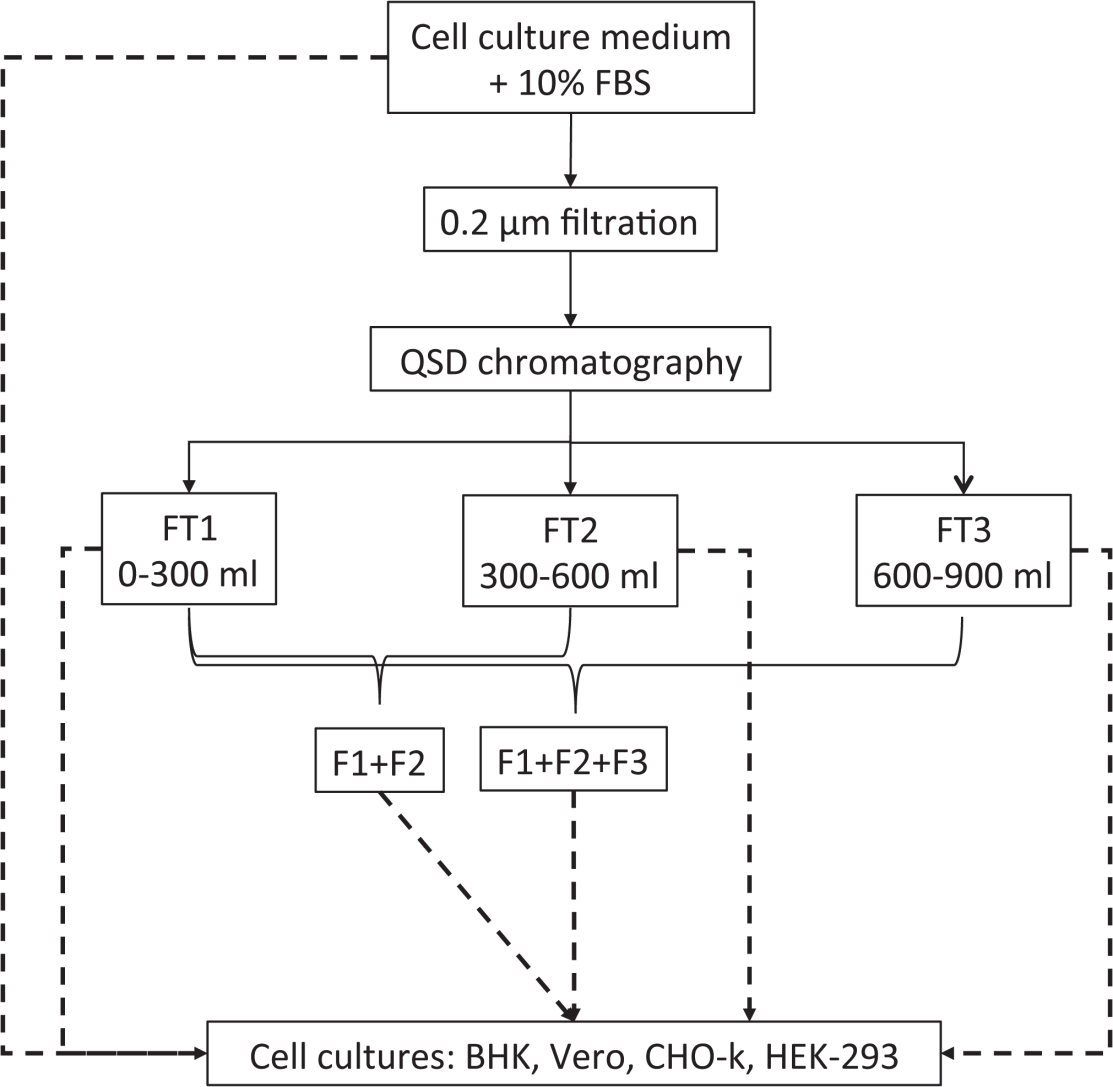
Experimental design for assessing the impact of the QSD filtration of growth media on cell growth. Three flow-through fractions (FT1, FT2, FT3) from the QSD column have been obtained and tested either individually, or after pooling (F1+F2; F1+F2+F3) to determine their capacity to support cell growth. Details of the procedure are described in the Material and Methods section.

The human embryonic kidney fibroblast (HEK293) cell line (ATCC CLR-1573; Invitrogen) was grown in DMEM, and the baby hamster kidney (BHK-21) cell line (ATCC CCL-10; Invitrogen) was grown in MEM. The African green monkey kidney epithelial (Vero) cell line (*Cercopithecus aethiops*; ATCC CCL-81; Invitrogen) was grown in MEM, and the Chinese hamster ovary epithelial (CHO-k1) cell line (ATCC CCL-61; Invitrogen) was grown in F-12K. The cell lines were maintained in sterile 10-cm culture dishes at 37°C in a controlled atmosphere containing 5% CO_2_. Sterile 24-well plates (Becton Dickinson, Franklin Lakes, USA) were seeded with 1 x 10^4^ cells/well, and allowed to adhere for 12 to 16 h. The cells were grown for 6 h in a serum-free medium, after which the medium was replaced with medium supplemented with 10% FBS. The cells were grown for < 5 days before being subjected to QSD filtration. The medium was replenished every 2 days during the growth period. Control cells were grown in medium supplemented with 10% FBS, but were not subjected to QSD filtration. Six culture conditions were used for each cell line as follows: control, F1, F2, F3, F1+F2, and F1+F2+F3. The experimental design used to assess the effect of the QSD filtration of the medium on cell growth and viability is presented in Fig 1.

#### Cell count and viability assay

Viable cells were counted for 5 days, using a Countess Automated Cell Counter (Invitrogen), according to the manufacturer instructions, and digital photographs were captured. Viability was determined using a 3-(4,5-dimethylthiazol-2-yl)-2,5-diphenyltetrazolium bromide (MTT) assay. Cells were seeded in 96-well plates at a density of 4 x 10^5^ cells/mL. The culture medium in each well was replaced with 100 μL of medium containing 500 μg/mL MTT, and the cells were cultured for an additional 4 h. One hundred microliters of dimethyl sulfoxide were added to each well to solubilize the tetrazolium product, and the absorbance of the contents of each well was measured at 540 nm by using a microplate reader. Cell viability was expressed as the percent of the absorbance of the control wells containing culture medium with 10% FBS. The data are reported as the mean ± standard deviation. The cell count data were analyzed using one-way analysis of variance. A P value of less than 0.05 was considered to indicate a statistically significant difference.

### PrP^Sc^ removal studies

#### Regulatory compliance

All of the experiments and assays in which the PrP^Sc^ was used were performed at the ViruSure research facilities (Donau City Strasse 1, A-1220, Vienna, Austria) in compliance with the “Principles of Good Laboratory Practice” published by the Organization for Economic Cooperation and Development in ENV/MC/CHEM(98)17 [19]; Part 58 of the United States Code of Federal Regulations Title 21 [20]; Austrian Good Laboratory Practices (Verordnung: GLP; BGBl. II Nr. 450/2006) [21]; and European Union regulations 2004/9/EC [22] and 2004/10/EC [23].

#### Start growth medium

The experiments used to assess QSD filtration for the removal of PrP^Sc^ were performed using DMEM supplemented with glutamine, 1 mM pyruvate, 0.1 mg/mL gentamicin, and 10% FBS (PAA, Linz, Austria; sourced from Australia).

#### PrP^Sc^ strain

The 263K strain of hamster-adapted scrapie was supplied as a 10% homogenate by Dr. Robert Rohwer of the Baltimore Research and Education Foundation (Baltimore, MD, USA). We passaged the strain by performing intracerebral inoculation on hamsters. The brains of affected hamsters were used to prepare a 10% homogenate in PBS. A microsomal/cytosolic fraction was prepared from the homogenate, as described previously [16], and stored at ≤ -60°C.

#### Interference testing

We investigated potential interferences in the detection of PrP^Sc^ in the samples. Materials were tested undiluted or at dilutions of 0.5 log_10_ or 1.0 log_10_, and spiked with the 263K strain to a final titer within approximately 2 log_10_ of the end point of the 263K stock used. The starting material was also tested as 10× and 50× concentrates. Following spiking, the samples were centrifuged at 15,558 × *g* for 60 min at 23°C ± 5°C. The supernatant was carefully removed, and the pellet was suspended in Tris-buffered saline containing 0.1% BSA (TBSA) in a volume 10- or 50-fold less than that of the original spiked sample. Proteinase K (PK) digestion and western blotting were performed using PK digestion in the presence of Triton X-100, as described previously [12]. The spiked process intermediates were compared with control spiked samples (spiked into TBSA) to identify evidence of interference with the detection of PrP^Sc^.

#### Spiking experiments and QyuSpeed D filtration

**Fig 2:**
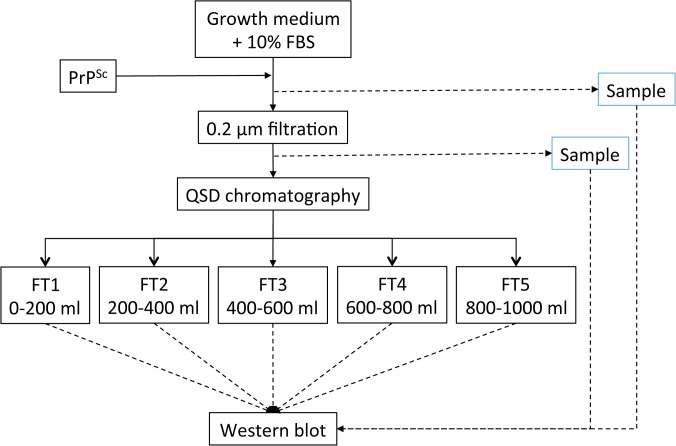
Experimental design for assessing the removal of PrP^Sc^ from growth media by using QSD filtration. Details of the procedure are described in the Material and Methods section.

All of the spiking and QSD filtration experiments were performed at 23°C ± 5°C. Complete growth medium (1100 mL) was passed through a 0.2-μm filter, and spiked with 1.0% 263K. The spiked medium was stirred for 5 min, and a 7.0-mL aliquot was stored at ≤ -60°C. The spiked starting material was passed through a 0.2-μm filter before use in the spiked runs to avoid loading aggregated PrP^Sc^ on the QSD column. A 7.0-mL aliquot of the prefiltrate was stored at ≤ -60°C. The QSD process was performed as described previously. The column was prewashed with 20 mL of equilibration buffer containing 20 mM Tris (pH 7.5) before 1000 ± 5 mL of spiked culture medium was loaded on the QSD column. The flow rate was reduced as the final 20% to 40% of the culture medium was loaded to maintain the recommended pressure of 0.2 MPa. Pressure and conductivity were monitored throughout the experiment. The filtrate was collected in five 200-mL fractions, and stored at ≤ -60°C. The process flow scheme and sample collection are presented in Fig 2.

#### Western blot analysis of PrP^Sc^

Samples were digested with PK to remove PrP^C^. After terminating the proteolytic reaction, the sample was mixed with SDS buffer, and boiled. The samples were diluted by 0.5 log_10_, and subjected to SDS-PAGE. The resolved protein bands were transferred to a PVDF membrane. The membrane was blocked, and probed using the 3F4 anti-PrP monoclonal antibody (Covance, Princeton, New Jersey, USA). The characteristic banding pattern of the 263K strain between 25 and 33 kDa was used to confirm the presence of the PrP^Sc^. The end point titer of the sample was the first dilution at which no PrP^Sc^ signal was observed on the western blot. The reduction factor (RF) was calculated following the Committee for Proprietary Medicinal Products (CPMP) “Note for Guidance on the Performance of Virus Clearance Studies” [24], as *RF* = (V_1_ x T_1_) _/_ (V_2_ x T_2_), where V_1_ and T_1_ are the volume and titer of the starting material, respectively, and V_2_ and T_2_ are the volume and titer of the filtrate fraction, respectively. In logarithmic terms, this equation can be expressed as log_10_[RF] = [log_10_ (V_1_) + log_10_(T_1_)]—[log_10_ (V_2_) + log_10_ (T_2_)]. The data were rounded to 3 decimal places throughout the calculations, except for the reduction factors, which were rounded to 2 decimal places.

## Results

### QyuSpeed D chromatography of growth media

**Fig 3:**
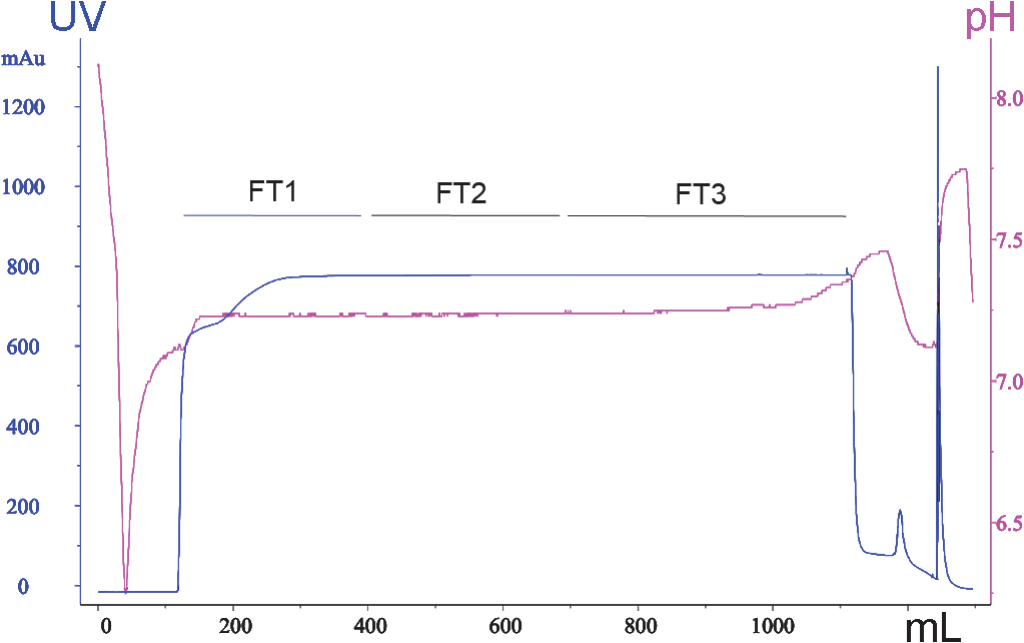
Chromatographic profile at 280 nm obtained when filtering DMEM medium supplemented with 10% FBS on the QSD device. The y-axis represents the absorbance at 280 nm (red line) and the y2 axis the pH value (blue line). The x-axis is the volume (mL) of growth medium passed through the QSD device. FT: flow-through. AU: absorbance unit

The chromatographic profile of the QSD filtration of DMEM with 10% FBS is shown in Fig 3. The optical density at 280 nm increased sharply after the medium was loaded on the column, indicating a high level of protein was detected. The 3 FTs were collected, and used for the cell culture experiments, as described previously. After the optical density of the filtrate returned to baseline, washing the column with high-conductivity buffer produced a peak in absorbance, indicating protein was eluted. The eluted fractions were collected for analysis. The chromatograms obtained for the filtration of MEM and F-12K media containing 10% FBS were similar.

### Growth medium analysis

**Fig 4:**
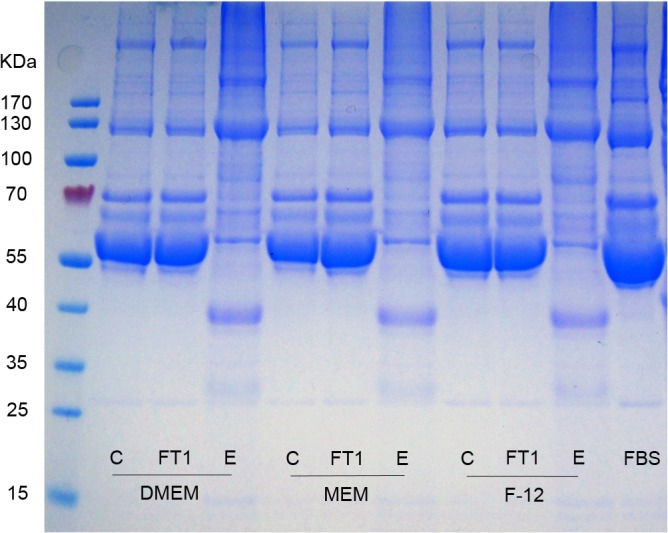
Sodium dodecyl sulfate electrophoresis under nonreducing conditions. Molecular weight protein markers (left lane); start growth medium (C); flow-through (FT) and eluate (E) of QSD-filtered FBS and growth media (DMEM, MEM, and F-12K) containing 10% FBS. Total protein content in each lane was 20 μg. kDa: kilodaltons

The SDS-PAGE analysis of the growth-media flow-through is shown in Fig 4 (data for FT2 and FT3 not shown). The flow-through fractions exhibited similar banding patterns to that of FBS. The eluates from all of the media contained several proteins present in FBS, 2 of which displayed apparent molecular weights of approximately 130 and 40 kDa. The chemical analysis revealed that QSD filtration had not substantially changed the chemical composition of the various filtrate fractions, compared with that of the respective starting material (Table 1).

**Table 1:** Chemical composition of growth media before (control) and in the flow-through 1 (FT1), 2 (FT2) and 3 (FT3) and eluate of QSD (N = 1; 5 fractions tested for each medium).

QSD	DMEM + 10% FBS					MEM + 10% FBS					F12 + 10% FBS				
	Control	FT1	FT2	FT3	Eluate	Control	FT1	FT2	FT3	Eluate	Control	FT1	FT2	FT3	Eluate
**Sample**	**1**	**2**	**3**	**4**	**5**	**6**	**7**	**8**	**9**	**10**	**11**	**12**	**13**	**14**	**15**
**Total Protein mg/mL**	0.4	0.4	0.4	0.4	0.4	0.4	0.4	0.4	0.4	0.3	0.4	0.4	0.4	0.4	0.4
**Glucose mg/dL**	467	470	475	470	7	110	109	109	107	1	189	192	194	190	4
**Chloride mEq/L**	138	139	140	141	550	142	143	141	140	707	151	152	151	151	797
**Sodium mEq/L**	176	178	180	181	519	164	166	163	163	659	167	168	167	167	743
**Potassium mEq/L**	6.8	6.9	7.0	7.0	4.5	6.7	6.8	6.7	6.7	4.6	4.5	4.5	4.5	4.5	4.2
**Calcium mg/dL**	8.5	8.0	8.3	8.1	0.1	5.7	4.9	7.4	5.2	0.0	2.3	2.6	2.5	2.5	0.1
**Phosphate mg/dL**	4.0	3.9	4.0	4.1	50.0	3.1	3.0	3.8	3.0	45.6	4.3	4.4	4.4	4.3	44.2
**Magnesium mg/dL**	2.2	2.2	2.3	2.2	0.0	2.0	2.0	2.1	2.0	0.0	1.7	1.7	1.7	1.7	0.1
**Iron μg/dL**	23	25	24	22	24	19	19	21	21	13	33	29	31	32	195
**Total Iron Binding Capacity μg/dL**	29	34	33	35	11	32	24	29	30	0	58	58	52	59	52
**Ferritin ng/mL**	1	1	1	1	1	1	1	1	1	1	1	1	1	1	1
**Vitamin B12 pg/mL**	<30.0	<30.0	<30.0	<30.0	478.9	<30.0	<30.0	<30.0	<30.0	332.0	>2000.0	>2000.0	>2000.0	>2000.0	>2000.0
**Folate ng/mL**	>20.00	>20.00	>20.00	>20.00	>20.00	>20.00	>20.00	>20.00	>20.00	>20.00	>20.00	>20.00	>20.00	>20.00	>20.00
**Hemoglobin g/dL**	0.2	0.1	0.2	0.2	0.1	0.2	0.1	0.2	0.2	0.3	0.0	0.0	0.0	0.0	0.0

### Cell culture data

**Fig 5:**
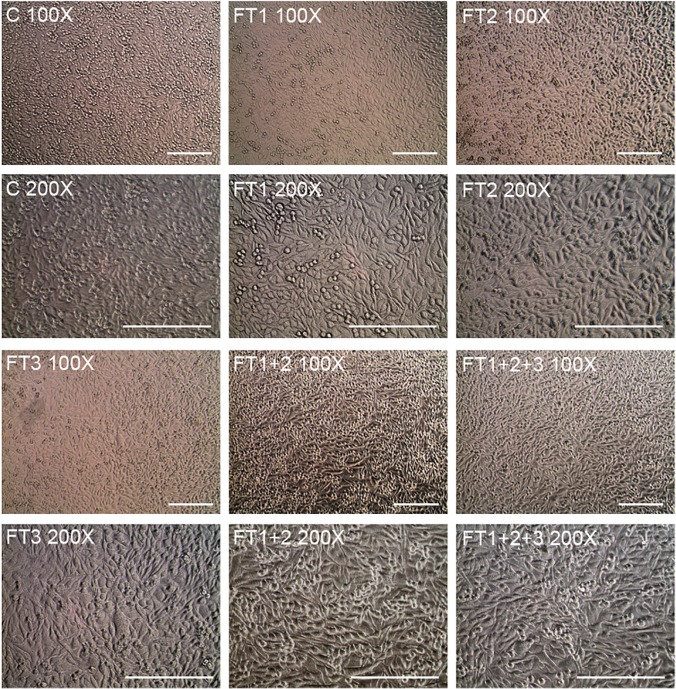
Morphology of HEK293 cells. Cells were grown in DMEM medium supplemented with 10% FBS without filtration (C) or following QSD filtration (FT1, FT2, FT3, FT1+FT2; FT1+FT2+FT3) FT: flow-through. The scale bar represents 100 μm.

**Fig 6:**
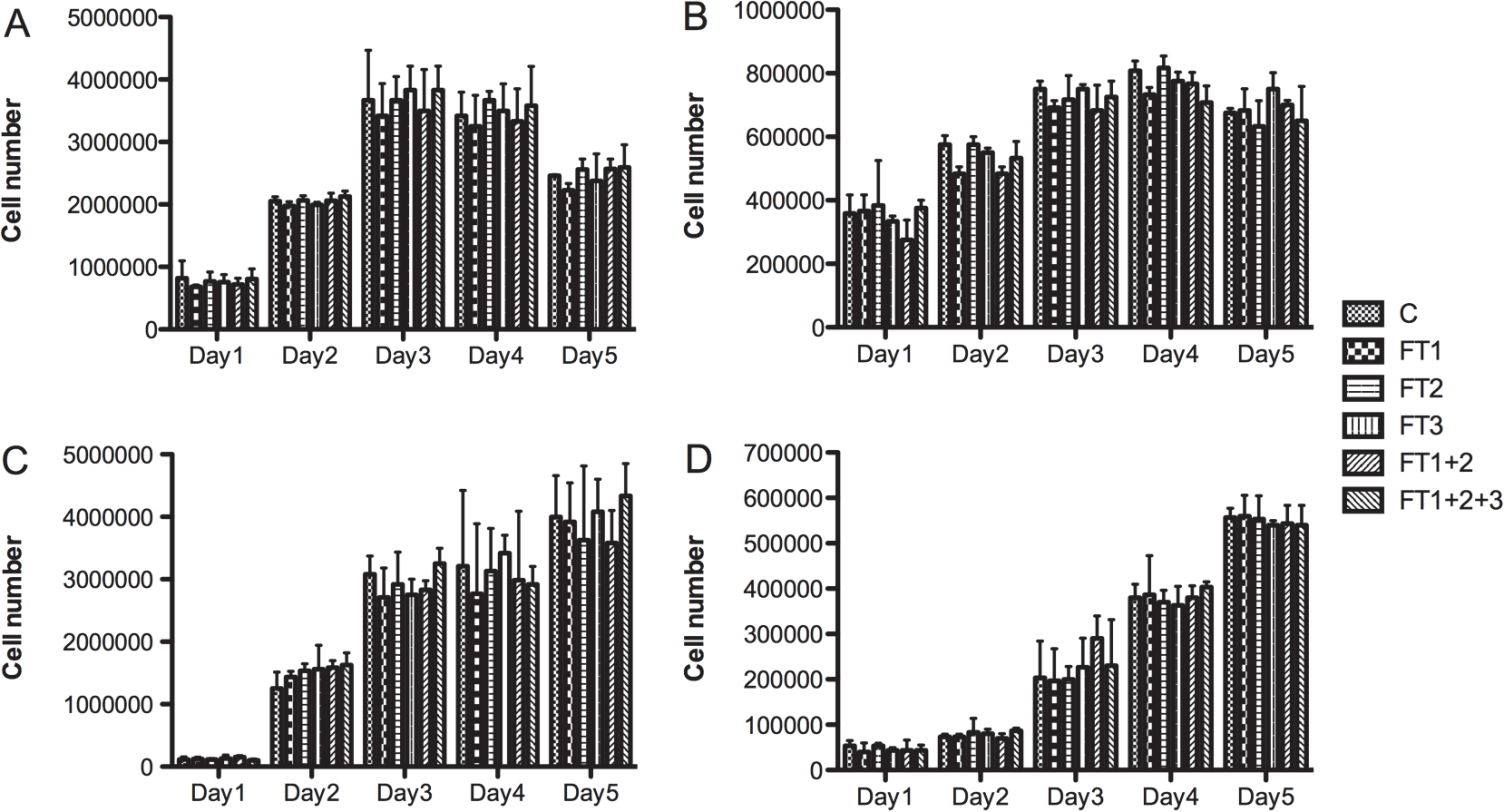
Cell count of BHK-21 (A), Vero (B), CHO (C), and HEK293 (D) cells. Cells were grown in DMEM medium supplemented with 10% FBS without filtration (C) or following QSD filtration (FT1, FT2, FT3, FT1+FT2; FT1+FT2+FT3). FT: flow-through. Mean +/- SD (N = 3)

**Fig 7:**
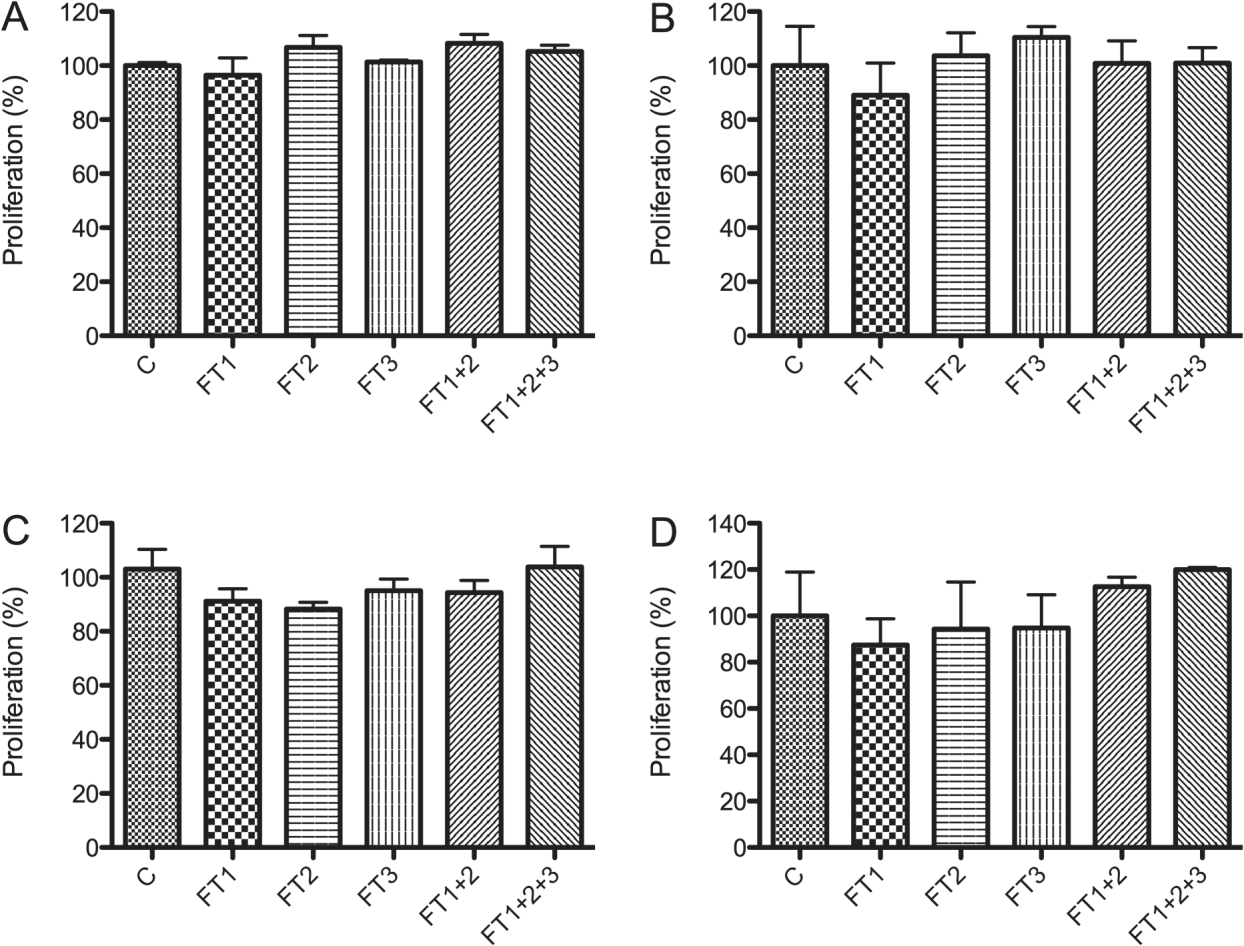
Cell viability assay at day 5 of BHK-21 (A), Vero (B), CHO (C), and HEK293 (D) cells. Cells were grown in DMEM medium supplemented with 10% FBS without filtration (C) or following QSD filtration (FT1, FT2, FT3, FT1+FT2; FT1+FT2+FT3). FT: flow-through. Mean +/- SD (N = 3).

No morphological differences were observed between the HEK293 cells grown in DMEM and those grown in the DMEM flow-through (Fig 5). Similar results were obtained for the other cell lines and media (data not shown). Cell count and viability data for all of the cell lines were not significantly different between the cells grown in the culture media and those grown in the respective media flow-through (Figs 6 and 7).

### PrP^Sc^ removal

**Fig 8:**
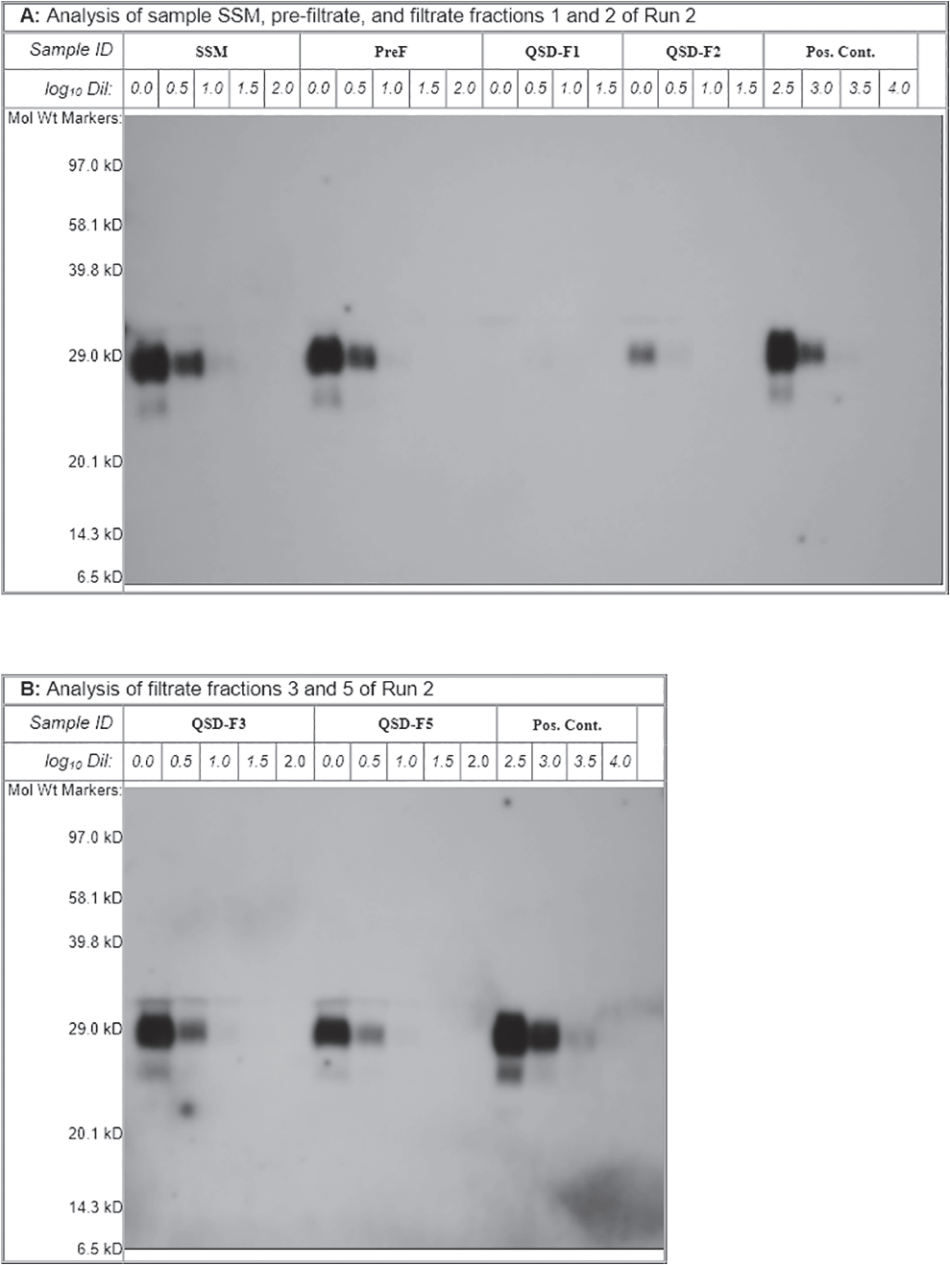
Western blotting of DMEM medium supplemented with 10% FBS and spiked with the 263K strain of scrapie. (A) Analysis of the spiked starting material, prefiltrate, and filtrate (flow through) fractions 1 and 2 of Run 2 (B) Analysis of filtrate fractions 3 and 5 of Run 2. MW: molecular weight; SSM: spiked start material; kDa: kilodalton; PreF: pre-filtrate sample; QSD-F1/F2/F3/F5: filtrate fractions 1/2/3/5; Pos. Cont.: Positive control. (N = 2).

We first verified whether the growth media interfered with PrP^Sc^ detection (data not shown). The DMEM with 10% FBS media was spiked with the 263K strain to a final titer within approximately 2 log_10_ of the end point of the scrapie stock used, and compared with the control spiked samples (spiked into TBSA) to identify evidence of interference in PrP^Sc^ detection by western blotting. The interference testing was performed using both undiluted DMEM 10%FBS and the same material diluted by 0.5 or 1.0 log_10_. To increase the sensitivity of the assay for processed samples, a 10× or 50× concentrated PrP^Sc^ sample prepared from the undiluted sample was also evaluated for potential interference. Because interference was observed for the 50× concentrated sample (greater than 1 log difference in titer when compared with the control- data not shown), the spiked starting material and prefiltrate were diluted by 0.5 log and the 10× concentration of the QSD filtrates were used to increase the limit of detection. The typical western blot patterns obtained using the 10x concentration procedure for the QSD-filtered DMEM with FBS are shown in Fig 8A and 8B (data presented are from Run 2). We obtained RF values of 2.5 log_10_ (Run 1) and ≥ 3.0 log_10_ (Run 2) for the first 200 mL of QSD-filtered growth medium, whereas the RF value of subsequent fractions decreased progressively to less than 2 log_10_ in the duplicate runs performed (Table 2). No significant loss of PrP^Sc^ was observed following prefiltration of the spiked medium by using the 0.2-μm filter (Fig 8A), indicating that the spike did not contain aggregated form of the PrP^Sc^ which would have otherwise been removed by such prefiltration.

**Table 2:** PrP^Sc^ (263K) reduction factors achieved in flow-through (FT) 1 to 5 during chromatography of DMEM growth medium containing 10% FBS on QSD device (duplicate experiments).

Samples	Run 1		Run 2	
	Log titer	RF	Log titer	RF
Starting filtered spiked material	4.30	**-**	4.30	**-**
0–200 mL flow-through (FT1)	1.80	**2.5**	≤1.30	**≥3.0**
200–400 mL flow-through (FT2)	2.30	**2.0**	2.30	**2.0**
400–600 mL flow-through (FT3)	2.80	**1.5**	2.80	**1.5**
600–800 mL flow-through (FT4)	2.80	**1.5**	2.80	**1.5**
800–1000 mL flow-through (FT5)	2.80	**1.5**	2.80	**1.5**

## Discussion

We used a 0.6-mL QSD adsorbent hollow-fiber anion-exchange chromatographic column to filter 1000 mL of 3 types of culture media without pressure build up. As expected, we observed the adsorption of FBS proteins on the QSD column, most of which had molecular weights of approximately 130 and 40 kDa. However, relatively small quantities of these proteins were lost, compared with their levels in the filtrate. The cell growth and viability characteristics of one human and three animal cell lines grown in the filtrated media were not significantly different from those of cells grown in the untreated media, which suggests that the proteins removed were quantitatively and qualitatively non-essential for cell growth. As detailed in European regulatory guidelines [14,24], we investigated whether this process had the ability to remove PrP^Sc^, thereby demonstrating that QSD filtration can improve the PrP^Sc^-related safety of growth media supplemented with FBS. The spiking experiments involving the use of the 263K hamster adapted scrapie strain and DMEM medium with FBS further demonstrated the capacity of QSD filtration for removing the PrP^Sc^ from solutions. Using a 0.6 mL-QSD column, we discovered that the QSD filtration of 200 mL of DMEM with FBS reduced the level of PrP^Sc^ in the filtrate by 2.5 to > 3 log_10_, compared with the level in the starting material.

The 263K strain of hamster-adapted scrapie [25] has been widely used in prion removal studies because it can replicate to high titers in the brains of hamsters. In contrast to PrP^C^, the endogenous nonpathogenic form of PrP, the PrP^Sc^ associated with TSE is more resistant to PK digestion, thus enabling the selective detection of the pathogenic PrP^Sc^. We prepared the 263K strain by isolating the microsomal/cytosolic fraction of hamster brain tissue using differential centrifugation to remove larger aggregates, leaving only the smaller microsomal membrane fragments in the supernatant. The PrP^Sc^ was detected using western blotting [25,26] following PK digestion. The reliability of PrP^Sc^ detection conducted using western blotting [13] correlates with that of biological measurements of PrP^Sc^ infectivity in rodent models [13,27–29]. As the 0.6 mL QSD column could remove 2.5 to > 3 log_10_ from 200 mL of growth medium containing 10 FBS, this implies that a 5-L QSD column should have the capacity to remove PrP^Sc^ from over 1650 L of such growth medium, making it cost-effective, especially as it is re-usable.

Although we did not determine the mechanism through which PrP^Sc^ was bound to the membrane of the QSD column, we speculate that it occurred through the interaction between the PrP^Sc^ and the hydrophobic polyglycidyl methacrylate backbone of the QSD membrane because hydrophobic interactions have been demonstrated to partition PrP^Sc^ during the fractionation of human plasma through the use of some depth filters [30,31]. Experimental spiking studies have also demonstrated that anion exchange resins used during plasma fractionation can remove significant amounts of PrP^Sc^, with typical reductions from 2 to > 3 log_10_ [25,30,32].

This study has 3 main limitations. First, we used the 263K strain of hamster-adapted scrapie in the spiking experiments. The efficiency of QSD filtration for the removal of PrP^Sc^ from other sources must be confirmed because each might have distinct biophysical properties [33]. Second, the infectivity of brain isolates of PrP^Sc^ was not compared directly to that of serum isolates. Thus, the use of PrP^Sc^ as a marker for infectivity in the growth media might not mimic infectious PrP^Sc^ [30]. However, the microsomal fractionation method that we used to isolate the PrP^Sc^ from hamster brain tissue has been used for preparing PrP^Sc^ spike preparations investigating prion removal from human plasma and products containing recombinant proteins expressed in cell culture [14]. Therefore, our spiked preparations likely contained infectious PrP^Sc^ that was identical or highly similar to that obtained from blood or plasma. Currently, all blood products can only be licensed within Europe following the submission of prion removal studies. These studies are almost exclusively performed by monitoring the partitioning of PrP^Sc^ as a marker of infectivity. There is currently no regulatory requirement to confirm data by e.g. western blot in a bioassay. The WB assay used in this manuscript, however, has been validated alongside an infectivity assay [12] providing additional assurances that the removal observed in this paper via Western blot would like be also seen in an infectivity assay. Third, we did not determine whether the efficiency of QSD filtration for the removal of PrP^Sc^ from DMEM is equivalent to that for removing PrP^Sc^ from MEM and F-12K media. However, the similar conductivity measurements of the filtrates obtained for the 3 types of media suggest the extent of PrP^Sc^ removal would likely be similar as well.

In conclusion, our experimental study revealed for the first time that a 0.6 mL hollow-fiber anion exchange chromatography can be used to remove 2.5 to > 3 log_10_ PrP^Sc^ from 200 mL of cell culture growth medium under conditions that can be implemented on a larger scale. A practical means of removing PrP^Sc^ from FBS is crucial because the β-sheet structure of the PrP^Sc^ contributes to aggregation and resistance to degradation by chemical and physical means [34], rendering prion inactivation unrealistic. Although nanofiltration, a method of virus removal widely used for plasma products, has been demonstrated to remove substantial amounts of PrP^Sc^ from solutions [30], the use of nanofiltration for filtering large volumes of growth medium would likely be impractical because of the expense. Therefore, QSD filtration is of practical value for numerous cell culture applications requiring growth media supplemented with FBS until alternatives are developed [35,36]. Finally, performing QSD filtration on the complete growth medium supplemented with FBS, rather than on FBS only, is a means to reduce the risks of adventitious contamination from any of the medium components.
